# Will the Too Prevalent Use of Plastics in Filling Teeth Work Injury to Operative Dentistry

**Published:** 1895-07

**Authors:** J. D. Patterson


ARTICLE IX.

### WILL THE TOO PREVALENT USE OF PLASTICS IN FILLING TEETH WORK INJURY TO OPERATIVE DENTISTRY.

BY J. D. PATTERSON, D. D. S.

This question is embodied in question No. 4 from the A. D. A. list of questions for the use of State and local societies for the present year. The full text of the question is as follows: "Is not operative dentistry liable to the same injury from the too prevalent use of plastic stoppings as occurred to prosthetic practice from the introduction of vulcanite?"

The framers of this question were, no doubt, somewhat hasty in formulating it, and it may not say just what they intended. We, however, get the gist of their intention, and I think it may be stated as follows: That the introduction of vulcanite cheapened prices and is so easily worked that a swarm of incompetents occupied the field heretofore filled by skillful metal-plate workers, and crowded them out, to the detriment of that field; and that the too prevalent use of plastics will also cheapen prices in the operative field, and will tend to push the skillful operator in gold filling out of business.

In the first place, I am inclined to deny the assumption that the introduction of vulcanite injured prosthetic dentistry. It did injure our profession generally, on account of the millions of serviceable perfect teeth that were sacrificed when cheap dentures were introduced. In this we find the injury, and, sad to say, this injury still continues to mar Nature's perfectness and to keep the dentist, in the esteem of popular opinion, upon a plane lower than his due. When the prosthetic dentist in placing dentures was limited to gold and platinum, the fees were beyond the reach of the majority, and so the people conserved their natural organs if they did not cause suffering. But upon the appearance of the cheap vulcanite, the natural organs, good, bad and indifferent, of the class formerly unable to pay the high fees, were sacrificed ruthlessly, and the accomplished men in the profession and the best informed patients have on this account deplored the advent of cheap vulcanite dentures. So far as vulcanite work is concerned, we are not willing to admit that the best results are accomplished more easily than with metal. True it is that in the usual slip-shod vulcanite work, commenced without artistic knowledge and completed with gross neglect of mechanical nicety, no particular skill and training is required; but I have always maintained that a denture of vulcanite, made in a superior manner, perfect anatomical form, perfect preparation and protection of model, perfect joints if in block teeth, or perfect similation of natural gum where block teeth are not used, all requires a degree of skill just as high as is required in making a gold plate. The result cannot be so perfect on account of the additional clumsiness of the vulcanite in its palatal portion, and lack of strength, but in securing the most perfect possible result with the despised vulcanite, your skill is as thoroughly tested with the one as with the other. It is true there is not required the skill in metal-working which the jewelry's apprentice knows about, but that knowledge can scarcely be held at a high valuation which brings only one dollar and seventy-five cents per diem to the finances of the competent journeyman jeweler.

For these reasons we cannot accept some of the evident conclusions of the question committee. Yet, understanding their intention to point to the consideration of whether the too prevalent use of plastics will work injury to operative dentistry, I will begin this part of the discussion by saying that for myself I do not believe it will. It cannot surely be denied that since the increased use of plastics, thousands of teeth are daily made useful that were formerly thought beyond the reach of dental art, and that also on account of the lessened cost of plastic materials thousands of people have blessed operative dentistry who would without the plastics never have known the relief of any operative procedure.

However we may boast of gold fillings and take pride in accomplishing beautiful results—as we all do—yet we must, I think, all have a feeling deep down in our reasoning and logical minds, that the filling of the future will be a plastic, that the ideal filling not yet found will be for the majority of cases a much-despised plastic; and can we deny that this very "too prevalent" use of the material will ultimately bring about great improvement in these same plastics, so that the ideal filling will thereby be more rapidly reached? We can look back only a few years and readily trace the improvement in nearly all of the plastic filling materials, and the improvement has been the natural result of an unusual demand, which our questioners would no doubt call a "too prevalent" use. It is possible poor judgment has been used in plastics, but poor judgment is also used with gold; but out of all mistakes we generally find the summing up somewhat nearer a better standard.

If it is desirable, as a few spirits in our profession seem to think, to exclude from the ranks all save a select coterie who scorn the plastics and obtain large fees with gold, who are continually emphasizing that they will not have amalgam in their offices, denominate it "nasty stuff" and say that it brings disgrace to our calling—the "too prevalent" use of plastics will, of course, infringe upon their assumed sovereign right and the circumsbribed coterie will be broken up; but when a larger and better horizon lightens up and the altruist dares to ask for the greatest results to the greatest number, this coterie will be found to be only squatter sovereigns after all. Our minds must be in sympathy with the larger patronage whose blessings are just as deserved as those whose relief from suffering could only be obtained with a full bank account.

The matter in a nutshell, it seems to me, is that, like working in vulcanite, we are too apt to believe that little or no skill is brought into demand with the employment of the plastics, when this is *positively incorrect.* The great skill in operative dentistry, in the first place, is in the adaptation of the right material; judgment of the pathological and other conditions of each case as it comes to hand; and then comes the manipulative dexterity. Now, as between gold and cement in filling, allowing that the two materials are used so as to secure for each the best result possible for each, and I cannot readily see where any greater skill is called into action in the case of gold work. It is more tedious, more exhaustive to work gold, and when the operation in gold is over, we point with pride to a great thing accomplished, but it is, I think, largely the exhaustion and relaxation of a manual labor, which comes after cutting and piling neatly a cord of wood. So far as the judgment and education and dexterity are involved, there is just as much of either used in the intelligent using of cement, only the exercise of these qualities is not so long continued.

If operative dentistry suffers from the use of the plastics in filling teeth, it will be because of the poor opinion of their value, which invariably leads to careless work in their use. Thus, in our opinion, if ill comes, it will lie with the ignorance and carelessness of dentists themselves, and is not due to the qualification and possibilities of the materials. Instead of decrying useful adjuncts, let us put the blame where it belongs and purge ourselves of blame in the matter.

As the plastics have widened our field of labor and made possible conservative operations once impossible, let us turn our attention, not to their obliteration, but to their improvement. Another consideration is this: the summit has been reached in the perfection of the various forms of gold, and we are not to expect that they will be improved; nor do we expect that new or improved methods of operating will come to be known. The skillful gold-filler of the future will never excel that of the present or of the last quarter of a century, but in plastic work we are constantly seeing improvement in materials and methods; and we confidently expect still more improvement in materials and methods, and we confidently expect still more improvement in the future. The advent of vulcanite did, as we have explained, have its evil as well as its good results, but we fail to see, in the light of the past, the present or a logical future, how operative dentistry is suffering or is going to suffer from the use of plastics. Their "too prevalent" use will only compel *more* prevalent visits to the dentist, which will give more constant supervision of the dental organs, and that is surely an unmixed evil to either patient or dentist.

In conclusion, the matter of art comes in. If any of our methods, or practices are inartistic; then those practices or methods should, if possible, be changed, for the dentist who is not an artist is a poor professional specimen. I apprehend that one of the most prominent changes in dental practice which we will see within a very short time will be a general movement with both patient and doctor, against the unnecessary placing of considerable surfaces of gold in the anterior teeth; which practice, many already see, is grossly inartistic and, we must admit, reminds us of the gold ring in the nose of the barbarian. In the correction of this objectionable practice, the plastics in filling must, it seems to me, be the principal factors. The observant practitioner who is fortunate in having among his patients the most cultured and artistic, cannot fail to have noticed their extreme repugnance to the appearance of gold in the mouth. They prefer the plastics and are glad to return frequently for their renewal rather than have flashing gold shock artistic feeling. We boast of our Western civilization, and I am as proud of it as any, but in many realms we must give preference to the judgment of people of places and countries older in artistic education and feeling. These people are almost to a unit against the gold display we have referred to. American dentists who have gone to practice in European capitals tell us that they almost forget how to insert gold, for their patients want none of it; first on account of its glaring prominence; and second because they object to the long-sittings often necessary for gold work. This latter feature is also yet another argument for the plastics, for there are few operators who have not witnessed considerable shock from protracted gold operations on account of reflex impressions made through the fifth nerve upon vital ganglia.

In summing up, I think we may say that the too prevalent use of plastics will not injure operative dentistry, but rather enlarge its field, because while ignorant, unskillful or indolent practitioners will injure by using it without judgment where a more permanent article should have been used, yet in the majority of cases even the "too prevalent" use of plastics will generally redound to the better comfort of humanity, and to the increased success of operative dentistry.—Proceedings of Kansas State Dental Association.—*Western Dental Journal.*

